# Malocclusion traits and oral health-related quality of life in adolescents: a multicenter cross-sectional study

**DOI:** 10.1093/ejo/cjag032

**Published:** 2026-05-19

**Authors:** Emma Göranson, Lillemor Dimberg, Aron Naimi-Akbar, Mikael Sonesson

**Affiliations:** Centre for Orthodontics and Pediatric Dentistry, Folktandvården Druvan, Region Östergötland County, Magasinsgatan 2, 601 82 Norrköping, Sweden; Department of Orthodontics, Faculty of Odontology, Malmö University, Carl Gustafs väg 34, 214 21 Malmö, Sweden; Health Technology Assessment Odontology (HTA-O), Faculty of Odontology, Malmö University, Carl Gustafs väg 34, 214 21 Malmö, Sweden; Swedish Agency for Health Technology Assessment and Assessment of Social Services, Box 61483, 102 33 Stockholm, Sweden; Health Technology Assessment Odontology (HTA-O), Faculty of Odontology, Malmö University, Carl Gustafs väg 34, 214 21 Malmö, Sweden; Department of Orthodontics, Faculty of Odontology, Malmö University, Carl Gustafs väg 34, 214 21 Malmö, Sweden

**Keywords:** malocclusion, oral health related quality of life, CPQ, PIDAQ, adolescent

## Abstract

**Aim:**

This study aimed to examine associations between separate malocclusion traits and oral health-related quality of life (OHRQoL) among adolescents with malocclusion. It also aimed to assess sex differences in OHRQoL and whether sex modified the associations between malocclusion traits and OHRQoL.

**Methods:**

We conducted a multicenter cross-sectional study among adolescents aged 12–19 years. Two groups were recruited: a no malocclusion group from general dentistry clinics and a malocclusion group from specialist orthodontic clinics. The participants completed two OHRQoL instruments—the generic Child Perceptions Questionnaire (CPQ_11–14_ short form) and the orthodontic-specific Psychosocial Impact of Dental Aesthetics Questionnaire (PIDAQ). Malocclusion was assessed based on intraoral photographs. Sociodemographic data were obtained from Statistics Sweden, and caries data from the national caries registry (SKaPa). Associations between malocclusion traits and OHRQoL were examined using linear regression models adjusted for confounders, with additional analyses of sex differences in OHRQoL and effect modification by sex.

**Results:**

The final sample included 103 without malocclusion and 541 with malocclusion. After adjustment, anterior crowding was associated with poorer OHRQoL compared with other malocclusion traits in both instruments. Among those with malocclusion, females reported poorer OHRQoL than males, whereas no sex differences were observed in the no malocclusion group. In the sex-stratified analyses, associations between separate malocclusion traits and OHRQoL were largely similar across sexes.

**Conclusions:**

Anterior crowding shows the strongest association with poorer OHRQoL. Females with malocclusion report poorer OHRQoL than males, but there is limited evidence of sex modifying associations between separate malocclusion traits and OHRQoL.

**Registration:**

ClinicalTrials.gov on 02 Sept 2021 (registration number: NCT05038865).

## Introduction

Malocclusions are common among children and adolescents. Approximately 70% present with some degree of malocclusion, while 35%–45% have a more severe condition and may benefit from orthodontic treatment [1, 2]. In Sweden and several other countries, orthodontic treatment of the most severe malocclusions in children and adolescents is included in public oral health care programs.

Untreated malocclusion negatively affects oral health-related quality of life (OHRQoL) [3, 4]. OHRQoL is ‘*a multidimensional construct that reflects (among other things) people’s comfort when eating, sleeping and engaging in social interaction; their self-esteem; and their satisfaction with respect to their oral health*’ [5].

OHRQoL is assessed using patient-reported outcome measures (PROMs). One of the most commonly used OHRQoL assessment instruments is the Child Perceptions Questionnaire (CPQ), which is a generic measure encompassing both a *symptoms/function* and a *well-being* domain. As malocclusion primarily affects the well-being domain and has limited impact on the symptoms/function domain, only part of the CPQ captures malocclusion-related effects, which may reduce its sensitivity to detect orthodontic-specific impacts [6–8]. In contrast, the Psychosocial Impact of Dental Aesthetics Questionnaire (PIDAQ) targets orthodontic-specific aspects of OHRQoL [9]. These aspects mainly reflect dental aesthetics and their psychosocial consequences. Using both generic and orthodontic-specific instruments in parallel may improve the ability to distinguish how malocclusion traits affect distinct dimensions of OHRQoL.

Previous research on the association between malocclusion and OHRQoL has largely relied on general population samples and dichotomous classifications of malocclusion, whereas fewer studies have focused on populations with malocclusion to examine differences between separate malocclusion traits and their relative impact on OHRQoL. While some studies have compared selected malocclusion traits, such as overjet or crossbite, they have been restricted to predefined malocclusion groups and younger age ranges, limiting their relevance for adolescents with more complex malocclusion profiles [10–12]. Moreover, multiple malocclusion traits frequently occur simultaneously, yet few studies account for this in their analyses.

Furthermore, the association between malocclusion and OHRQoL may be influenced by several confounding factors, that is, variables affecting both malocclusion and OHRQoL.

Despite similar overall malocclusion prevalence in males and females, specific malocclusion traits exhibit sex-specific differences [1, 2], and females are consistently overrepresented in orthodontic populations [13–15]. This discrepancy is commonly attributed to sex differences in self-perceived treatment need and dental dissatisfaction, which may influence both care-seeking behavior and impacts on OHRQoL [16, 17]. In addition to sex, age represents an important potentially confounding demographic factor, as the presence of malocclusion, as well as its perception and psychosocial impact, may vary across adolescence [18, 19].

Socioeconomic factors may not strongly influence the overall prevalence of malocclusion but is associated with several upstream factors—such as caries and access to interceptive orthodontic treatment—that may affect the development and severity of specific malocclusion traits [20, 21]. Socioeconomic factors appear to influence access to care, as adolescents with low socioeconomic status are less likely to receive orthodontic treatment—even within publicly funded systems [13, 15]. Furthermore, low socioeconomic status impairs OHRQoL, and socioeconomic factors may influence how malocclusion affects OHRQoL [22–24].

Dental caries represents another possible confounder. Caries is a major cause of premature tooth loss, which increases the risk of malocclusion in the permanent dentition [20]. Also, untreated caries has a well-established negative impact on OHRQoL [23, 25–27].

Taken together, these pathways illustrate the complex relationship between malocclusion and OHRQoL and underscore the need to measure and account for potential confounders—such as demographics, socioeconomic factors, and caries—in the analyses.

### Aims

This study aims to evaluate the associations between separate malocclusion traits and OHRQoL among adolescents with malocclusion, while accounting for co-existing malocclusion traits and relevant confounders. It also aims to explore sex differences in OHRQoL and to assess whether sex modifies the associations between separate malocclusion traits and OHRQoL.

## Methods

### Study design and setting

This multicenter cross-sectional study was conducted in Sweden at six general dental clinics in Region Östergötland and five specialist orthodontic clinics: Folktandvården Eastmaninstitutet Stockholm; the Department of Orthodontics at the Faculty of Odontology, Malmö University; and the three orthodontic clinics in Region Östergötland (Motala, Norrköping, and Linköping).

### Participants

Eligible participants were adolescents aged 12–19 years attending the study clinics. Recruitment was structured to include two predefined groups: a no malocclusion group and a malocclusion group. Adolescents attending routine dental examinations at general dentistry clinics with an Index of Orthodontic Treatment Need—Dental-Health Component (IOTN-DHC) grade of 1 or 2 were included in the *no malocclusion group*. Patients attending pretreatment consultations at specialist orthodontic clinics with an IOTN-DHC grade of 3, 4 or 5 constituted the *malocclusion group* [28].

Exclusion criteria for both groups were American Society of Anesthesiologists (ASA) physical status class III or higher and previous or ongoing comprehensive orthodontic treatment (including fixed appliances or aligners). Patients with previous interceptive orthodontic treatment were not excluded.

### Study procedure

Between June 2021 and December 2023, potentially eligible adolescents were consecutively approached during scheduled visits at the participating clinics. After written informed consent had been obtained, participants completed the OHRQoL questionnaires at the clinic, with the opportunity to ask questions. This was followed by standardized intraoral photographic documentation (five views: frontal, right and left buccal, maxillary occlusal, and mandibular occlusal).

Participants were excluded from the final analytical sample if photographic assessment indicated nonfulfillment of group-specific eligibility criteria, fractured or severely discolored maxillary incisors, or more than 10 missing questionnaire responses.

### Measures

#### OHRQoL measures

OHRQoL was measured with two instruments that have previously been validated in Swedish: the CPQ for children 11–14 years, Impact Short Form, 16 items (CPQ_11–14_–ISF:16) and the PIDAQ [9, 29–32]. The CPQ includes 16 items scored on a 5-point scale Likert scale (0–4), yielding a total score of 0–64 across two domains: *symptoms/function* (items 1–8) and *well-being* (items 9–16). Two additional global items assess overall impact and are not included in the total score [32]. PIDAQ also uses a 5-point Likert scale for response options but contains 23 items summing up to a total score of 0–92. The Swedish version of PIDAQ demonstrates two domains: *dental esthetic self-confidence* (items 1–7, 18, and 20–23) and *psychosocial impact* (items 8–17 and 19) [31]. To minimize order effects, the order of questionnaire administration was determined using block randomization (block size = 10).

#### Malocclusion assessment

Malocclusion was initially assessed using the IOTN-DHC to verify group eligibility, as described above. The assessment was based on the intraoral photographs supplemented with information from dental records, including overjet measurements, lip competence, functional mandibular shifts, and dental radiographs.

All IOTN-DHC assessments were performed by a single trained and calibrated investigator (EG) [32]. To assess intra-rater reliability, the IOTN-DHC classification was repeated after a minimum interval of 14 days in 30 consecutive cases.

In addition to overall malocclusion severity, separate malocclusion traits were assessed for the malocclusion group using the same photographic and record-based material. These traits were categorized as follows:

- **Overjet** was measured in millimeters as the horizontal distance between the labial surface of the mandibular incisor and the incisal edge of the corresponding maxillary incisor, parallel to the occlusal plane in intercuspal position; the maximum value was recorded. Measurements were primarily obtained from dental records and, when unavailable, estimated from the photos. Overjet was classified as normal (−1 to 6 mm), increased (>6 mm), or reverse (<−1 mm).
- **Overbite** was assessed from the photos and categorized as normal vertical overlap, open bite (>2 mm), or deep bite (complete coverage of the mandibular incisors). Analyses were based on visual estimation from the photos.
- **Posterior crossbite** was assessed from photos and defined as lingual or buccal occlusion of premolars or molars. Analyses were based on visual estimation from the photos.
- **Anterior crowding** was defined as a lack of space exceeding 2 mm in the incisor–canine region (teeth 13–23 and/or 33–43) and dichotomized as present or absent, with presence defined as crowding in the maxilla, mandible, or both. Analyses were based on visual estimation from the photos.
- **Anterior spacing** was defined as the presence of at least one visible interdental space >2 mm in the anterior segment (incisor–canine region; teeth 13–23 and/or 33–43). Analyses were based on visual estimation from the photos.
- **Impacted teeth** were defined based on diagnoses recorded in the patient dental records and third molars were excluded.

#### Demographic and socioeconomic data

Demographic data (sex, year of birth, country of origin, and municipality type) and socioeconomic data (parental income and education) were obtained from Statistics Sweden (SCB). Parental data were collected from the biological mother and father, or, when applicable, from adoptive parents. Caries data (decayed and filled teeth, DFT) were retrieved from the Swedish Quality Registry for Caries and Periodontal Disease (SKaPa). SCB linked participants across the registries using the personal identification number [33]. The datasets were anonymized before delivery to the research group.

### Statistical analysis

The data analysis was performed in Stata v.19.1 software (Stata Corp LLC, College Station, TX, USA). For questionnaires with 1–10 missing responses (calculated across both questionnaires combined), median imputation was performed within each respective questionnaire, as detailed in Supplementary Table S1. Intra-rater reliability of malocclusion measurements was evaluated with Cohen’s Kappa, interpreted according to Landis and Koch [34]. The data was summarized with descriptive statistics. Distributions of OHRQoL scores were visualized using boxplots. Sex differences within the malocclusion and no malocclusion groups were analysed using independent-samples *t*-tests. Linear regression models were used to examine associations between malocclusion traits and OHRQoL, including unadjusted models and multivariable models adjusted for relevant confounders. In addition, stratified analyses by sex were performed in the adjusted models to explore potential effect modification, and interaction by sex was formally tested. Confounder selection was informed by a directed acyclic graph (DAG), constructed based on existing literature and subject-matter knowledge [35]. The DAG is provided in Supplementary Figure S1. Statistical significance was defined as *P* < 0.05.

### Ethical considerations

The study protocol and informed consent form were approved by the Swedish Ethical Review Authority (ref. no. 2020-05319 with an amendment approved under ref. no. 2023-03152-02). Prior to enrollment, written informed consent was obtained from all participants. For those younger than 15 years, consent was additionally obtained from a parent or legal guardian.

### Reporting standards

The study was reported following the Strengthening the Reporting of Observational studies in Epidemiology (STROBE) guidelines [36].

## Results

### Participant characteristics

**Figure 1:**
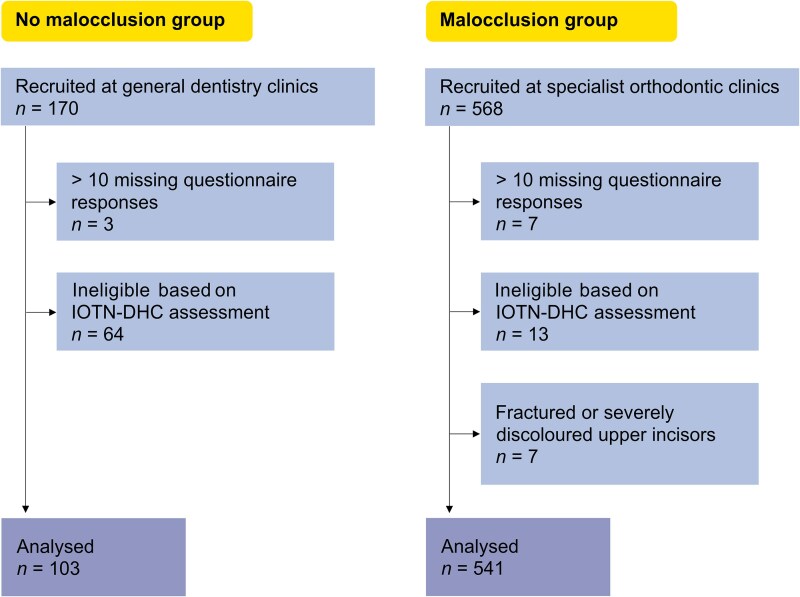
Flow chart of participant selection.

The final sample consisted of 103 participants in the no malocclusion group and 541 participants in the malocclusion group. The recruitment and exclusion process are summarized in Fig. 1. Baseline demographic, socioeconomic, and caries characteristics of participants with and without malocclusion are presented in Table 1. Supplementary Table S1 provides a detailed description of each variable and the data cleaning procedure.

**Table 1:** Demographic, socioeconomic, and dental-health characteristics by malocclusion status.

Characteristics	Variables	No malocclusion (*n* = 103)	Malocclusion (*n* = 541)
Demographics	Sex		
	Males	57 (55.3)	219 (40.5)
	Females	46 (44.7)	322 (59.5)
	Age group		
	12–15 years	30 (29.1)	230 (42.5)
	16–19 years	73 (70.9)	311 (57.5)
	Migration background		
	Swedish background	58 (56.3)	428 (79.1)
	Foreign background	45 (43.7)	113 (20.9)
	Municipality type		
	Urban	88 (85.4)	302 (55.9)
	Nonurban	15 (14.6)	238 (44.1)
Socioeconomics	Parental income		
	Low income	41 (40.2)	159 (29.4)
	Middle/high income	61 (59.8)	381 (70.6)
	Parental education		
	Primary/lower secondary	9 (8.9)	21 (3.9)
	Upper secondary	35 (34.7)	140 (25.9)
	University/college	57 (56.4)	379 (70.2)
Caries	DFT		
	0	42 (40.8)	295 (56.5)
	1–4	52 (50.5)	204 (39.1)
	≥5	9 (8.7)	23 (4.4)

Values are *n* (%). DFT, decayed, filled teeth. Column numbers may not equal the total sample (=644) due to missing data. Percentages may not sum to 100% due to rounding.

### Malocclusion assessment

The intra-rater reliability of IOTN-DHC grading was substantial, with 86.7% observed agreement and a Cohen’s kappa of 0.76 (*P* < 0.001).

The malocclusion traits in the malocclusion groups are presented in Table 2. Anterior crowding was the most frequently occurring malocclusion trait with a prevalence of 76%. Impacted teeth included both anterior and posterior teeth, with most cases being anterior (35/40).

**Table 2:** Frequency of malocclusion traits in the malocclusion group (*n* = 541).

Malocclusion trait	Category	*n* (%)
Overjet	Normal (−1 to 6 mm)	373 (69.0)
	Increased (>6 mm)	154 (28.5)
	Reverse (<−1 mm)	14 (2.6)
Overbite	Normal vertical	472 (87.3)
	Open bite (>2 mm)	9 (1.7)
	Deep bite (fully covered)	60 (11.1)
Posterior crossbite	No	408 (75.4)
	Yes	133 (24.6)
Anterior crowding	No	130 (24.0)
	Yes	411 (76.0)
Anterior spacing	No	461 (85.2)
	Yes	80 (14.8)
Impacted teeth	No	501 (92.6)
	Yes	40 (7.4)

Malocclusion traits are not mutually exclusive.

### OHRQoL scores by malocclusion status, malocclusion traits, and sex

The no malocclusion group had a mean CPQ total of 10.3 (SD 6.4) and a mean PIDAQ total of 20.5 (SD 17.2). The malocclusion group had mean CPQ and PIDAQ scores of 17.2 (SD 10.2) and 48.0 (SD 23.8), respectively. Results for separate malocclusion trait and CPQ and PIDAQ domains are presented in Table 3.

**Table 3:** Mean OHRQoL (CPQ and PIDAQ) scores by malocclusion status and separate malocclusion traits.

		*n*	CPQ				PIDAQ		
			Global	Total	Symptoms	Well-being	Total	Self-Conf	Psychosocial
No malocclusion		103	3.0 (1.5)	10.3 (6.4)	6.9 (3.5)	3.4 (4.2)	20.5 (17.2)	14.9 (10.1)	5.6 (8.1)
Malocclusion		541	3.4 (1.7)	17.2 (10.2)	8.4 (4.4)	8.8 (7.4)	48.0 (23.8)	32.7 (12.1)	15.3 (13.2)
*Among individuals with malocclusion*									
Overjet	Normal (−1 to 6 mm)	373	3.4 (1.7)	17.0 (10.4)	8.4 (4.5)	8.6 (7.5)	47.7 (24.2)	32.4 (12.4)	15.3 (13.3)
	Increased (>6 mm)	154	3.5 (1.6)	18.1 (9.6)	8.6 (4.4)	9.5 (7.2)	49.7 (23.0)	33.8 (11.1)	15.9 (13.2)
	Reverse (<−1 mm)	14	2.9 (1.8)	12.9 (8.1)	7.1 (3.1)	5.8 (5.5)	36.0 (18.2)	28.6 (13.8)	7.4 (5.9)
Overbite	Normal vertical	472	3.4 (1.7)	17.6 (10.2)	8.5 (4.4)	9.1 (7.5)	49.1 (23.7)	33.3 (11.9)	15.9 (13.3)
	Open bite (>2 mm)	9	3.3 (1.0)	14.9 (6.4)	7.8 (2.6)	7.1 (5.0)	42.1 (19.9)	30.1 (12.0)	12.0 (9.2)
	Deep bite (fully covered)	60	3.0 (1.4)	14.9 (9.9)	8.3 (4.5)	6.5 (6.7)	39.7 (23.6)	28.7 (13.0)	10.9 (12.0)
Posterior crossbite	No	406	3.4 (1.7)	17.3 (10.3)	8.4 (4.4)	9.0 (7.5)	48.5 (24.0)	33.0 (12.1)	15.6 (13.2)
	Yes	133	3.5 (1.6)	17.0 (9.9)	8.7 (4.5)	8.3 (7.1)	46.3 (23.3)	31.9 (12.0)	14.4 (13.0)
Anterior crowding	No	130	3.2 (1.7)	15.1 (10.0)	7.6 (4.2)	7.4 (7.6)	40.8 (24.4)	28.5 (13.1)	12.2 (12.6)
	Yes	411	3.5 (1.6)	17.9 (10.1)	8.7 (4.5)	9.2 (7.3)	50.3 (23.2)	34.0 (11.5)	16.2 (13.2)
Anterior spacing	No	461	3.4 (1.6)	17.3 (10.2)	8.6 (4.5)	8.7 (7.3)	47.8 (23.7)	32.6 (12.1)	15.2 (13.1)
	Yes	80	3.4 (1.9)	17.0 (9.9)	7.7 (4.1)	9.4 (7.9)	49.0 (24.6)	33.2 (12.0)	15.8 (13.8)
Impacted teeth	No	501	3.5 (1.6)	17.6 (10.1)	8.5 (4.4)	9.1 (7.4)	49.1 (23.6)	33.3 (11.8)	15.8 (13.2)
	Yes	40	2.6 (1.6)	12.3 (9.3)	7.2 (4.0)	5.2 (6.5)	34.2 (22.5)	25.5 (14.0)	8.7 (10.4)

Values are mean (SD). CPQ, Child Perceptions Questionnaire (CPQ_11–14_–ISF:16); CPQ Global, (range 0–8); CPQ Total, total score (range 0–64); CPQ Symptoms, symptoms/function domain (range 0–32); CPQ Well-being, well-being domain (range 0–32). PIDAQ, Psychosocial Impact of Dental Aesthetics Questionnaire; PIDAQ Total, total score (range 0–92); PIDAQ Self-Conf, dental esthetic self-confidence domain (range 0–48); PIDAQ Psychosocial, psychosocial impact domain (range 0–44).

**Figure 2:**
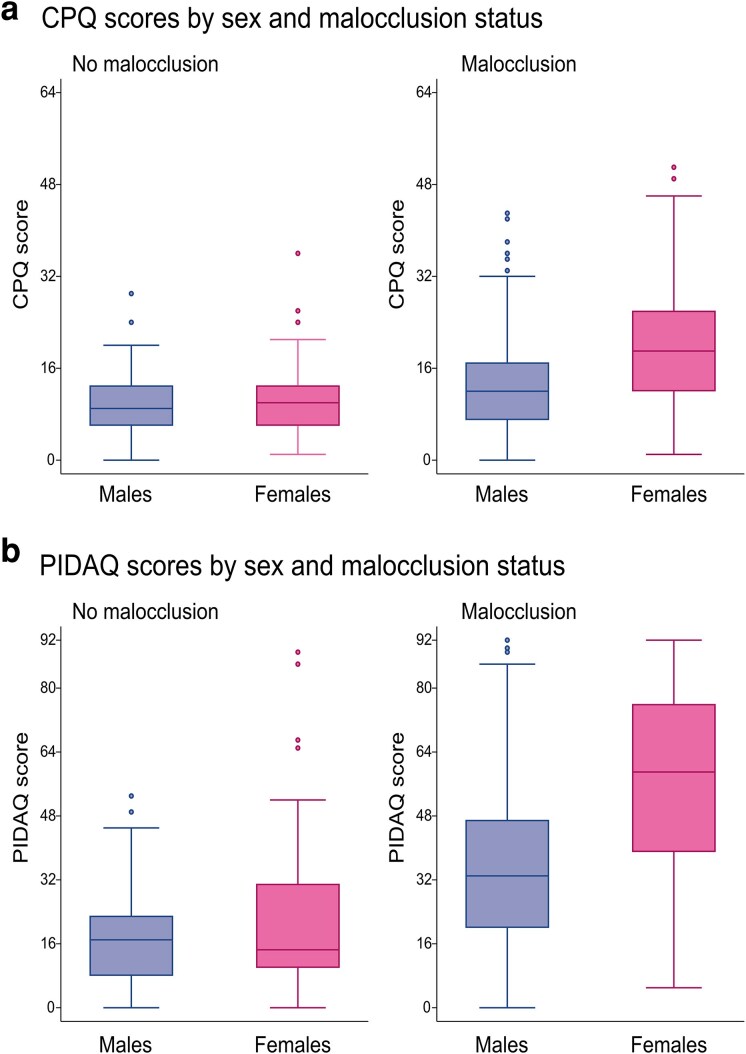
Box plots of (a) CPQ and (b) PIDAQ total scores by sex and malocclusion status.

In the no malocclusion group, no significant differences were observed between sexes for either total scores, global questions or domains. In contrast, in the malocclusion group, all domains and both total scores indicated poorer OHRQoL among females compared with males (Table 4). The differences are illustrated in Fig. 2.

**Table 4:** Mean OHRQoL (CPQ and PIDAQ) scores by malocclusion status and sex.

	*n*	CPQ				PIDAQ		
		Global	Total	Symptoms	Well-being	Total	Self-Conf	Psychosocial
No malocclusion								
Boys	57	3.1 (1.5)	10.0 (5.7)	6.8 (3.4)	3.2 (3.7)	18.8 (13.1)	14.0 (8.1)	4.7 (6.0)
Girls	46	2.9 (1.6)	10.8 (7.2)	7.0 (3.7)	3.7 (4.8)	22.6 (21.2)	15.9 (12.1)	6.7 (10.0)
Sex difference (*P*)		0.469	0.538	0.756	0.502	0.262	0.361	0.211
Malocclusion								
Boys	219	3.2 (1.6)	13.2 (8.8)	7.6 (4.2)	5.6 (6.0)	35.4 (20.1)	27.2 (11.8)	8.2 (10.0)
Girls	322	3.5 (1.7)	20.0 (10.1)	9.0 (4.5)	11.0 (7.5)	56.5 (22.4)	36.4 (10.8)	20.1 (12.9)
Sex difference (*P*)		0.016	<0.001	<0.001	<0.001	<0.001	<0.001	<0.001

Values are mean (SD). Sex differences were assessed using independent-samples *t*-tests. CPQ, Child Perceptions Questionnaire; CPQ Total, total score (range 0–64); CPQ Symptoms, symptoms/function domain (range 0–32); CPQ Well-being, well-being domain (range 0–32). PIDAQ, Psychosocial Impact of Dental Aesthetics Questionnaire; PIDAQ Total, total score (range 0–92); PIDAQ Self-Conf, dental esthetic self-confidence domain (range 0–48); PIDAQ Psychosocial, psychosocial impact domain (range 0–44).

### Association between malocclusion traits and OHRQoL

In unadjusted analyses, anterior crowding was associated with poorer OHRQoL than other malocclusion traits, as assessed with both CPQ (*β* = 2.9; CI 0.9–4.9) and PIDAQ (*β* = 9.5; CI 4.8–14.1). Deep bite and impacted teeth were associated with better OHRQoL, particularly for PIDAQ.

After adjustment for age, sex, and co-occurring malocclusion traits, anterior crowding remained significantly associated with poorer OHRQoL in both instruments (CPQ *β* = 3.6, CI 1.1–6.0; PIDAQ *β* = 11.5, CI 6.2–16.9). Spacing was associated with poorer OHRQoL when assessed with PIDAQ (*β* = 11.1, CI 4.9–17.3). Reverse overjet was associated with better OHRQoL when assessed with PIDAQ (*β* = −11.9; CI −23.6 to −0.2), while the CPQ analyses showed estimates in the same direction but did not reach statistical significance. For impacted teeth, the adjusted estimates indicated better OHRQoL, but the associations were not statistically significant (Table 5). Supplementary Figure S2 provides an illustration of the adjusted results.

**Table 5:** Linear regression analyses of malocclusion traits and OHRQoL (CPQ and PIDAQ) scores among individuals with malocclusion.

	CPQ total				PIDAQ total			
	Unadjusted models^a^		Adjusted models^b^		Unadjusted models^a^		Adjusted models^b^	
	*β* (95% CI)	*P*	*β* (95% CI)	*P*	*β* (95% CI)	*P*	*β* (95% CI)	*P*
Overjet								
*Normal (−1 to 6 mm) (ref)*								
Increased (>6 mm)	1.1 (−0.9–3.0)	0.274	1.6 (−0.3–3.5)	0.092	2.0 (−2.5–6.4)	0.386	3.6 (−0.5–7.8)	0.086
Reverse (<−1 mm)	−4.2 (−9.6–1.2)	0.130	−4.6 (−9.9–0.8)	0.093	−11.7 (−24.4–1.0)	0.070	−11.9 (−23.6 to −0.2)	0.046
Overbite								
*Normal vertical (ref)*								
Open bite (>2 mm)	−2.7 (−9.4–4.0)	0.430	−1.2 (−7.6–5.2)	0.708	−7.0 (−22.7–8.6)	0.218	−4.2 (−18.3–9.8)	0.555
Deep bite (fully covered)	−2.7 (−5.4–0.0)	0.053	0.2 (−2.5–2.9)	0.897	−9.5 (−15.9 to −3.1)	0.004	−1.3 (−7.1–4.6)	0.672
Posterior crossbite								
*No (ref)*								
Yes	−0.4 (−2.4–1.6)	0.717	0.3 (−1.6–2.2)	0.753	−2.3 (−7.0–2.4)	0.338	−0.4 (−4.6–3.9)	0.866
Anterior crowding								
*No (ref)*								
Yes	2.9 (0.9–4.9)	0.005	3.6 (1.1–6.0)	0.004	9.5 (4.8–14.1)	<0.001	11.5 (6.2–16.9)	<0.001
Anterior spacing								
*No (ref)*								
Yes	−0.2 (−2.7–2.2)	0.847	2.3 (−0.5–5.1)	0.112	1.2 (−4.5–6.8)	0.685	11.1 (4.9–17.3)	<0.001
Impacted teeth								
*No (ref)*								
Yes	−5.3 (−8.5 to −2.0)	0.002	−2.0 (−5.2–1.2)	0.225	−14.9 (−22.4 to −7.3)	<0.001	−7.0 (−14.1–0.0)	0.050

Intercepts omitted from the table for clarity. CPQ, Child Perceptions Questionnaire (CPQ_11–14_–ISF:16); PIDAQ, Psychosocial Impact of Dental Aesthetics Questionnaire. ^a^Univariable linear regression. Analyses were performed on the total malocclusion group (*n* = 541). ^b^Multivariable linear regression, adjusted for other malocclusion traits, sex, age group, country of origin, municipality type, parental income, parental education, and caries. Analyses restricted to individuals with malocclusion and complete data (*n* = 520).

### Sex-stratified analyses

In the sex-stratified adjusted analyses (Table 6), associations between malocclusion traits and OHRQoL were largely similar in males and females with few exceptions. When OHRQoL was assessed using CPQ, a significant interaction with sex was observed (interaction *P* = 0.03), with spacing associated with poorer OHRQoL compared with other malocclusion traits among females but not among males. Using PIDAQ, both crowding and spacing were associated with impaired OHRQoL relative to other malocclusion types among females but not among males; however, no statistically significant interaction with sex was observed (interaction *P* = 0.19).

**Table 6:** Adjusted linear regression analyses of the association between malocclusion traits and OHRQoL (CPQ and PIDAQ) scores among individuals with malocclusion, stratified by sex.

	CPQ total				PIDAQ total			
	Boys		Girls		Boys		Girls	
	*β* (95% CI)	*P*	*β* (95% CI)	*P*	*β* (95% CI)	*P*	*β* (95% CI)	*P*
Overjet								
*Normal (−1 to 6 mm) (ref)*								
Increased (>6 mm)	1.9 (−0.6–4.4)	0.133	1.2 (−1.5–3.9)	0.396	2.0 (−4.1–8.1)	0.520	4.5 (−1.2–10.2)	0.119
Reverse (<*−*1 mm)	−4.9 (−10.9–1.1)	0.108	−2.9 (−12.3–6.5)	0.548	−8.8 (−23.4–5.8)	0.235	−12.8 (−32.6–7.0)	0.204
Overbite								
*Normal vertical (ref)*								
Open bite (>2 mm)	−2.0 (−11.2–7.1)	0.663	−1.9 (−11.4–7.6)	0.695	2.4 (−19.7–24.5)	0.829	−6.8 (−26.7–13.1)	0.503
Deep bite (fully covered)	−0.4 (−3.6–2.7)	0.795	−0.2 (−4.5–4.0)	0.925	0.3 (−7.4–7.9)	0.947	−5.5 (−14.4–3.5)	0.229
Posterior crossbite								
*No (ref)*								
Yes	0.1 (−2.5–2.6)	0.959	1.4 (−1.4–4.2)	0.318	−2.1 (−8.2–4.0)	0.493	2.8 (−3.1–8.6)	0.349
Anterior crowding								
*No (ref)*								
Yes	2.8 (−0.2–5.7)	0.070	3.1 (−0.7–6.9)	0.106	5.8 (−1.5–13.0)	0.118	16.1 (8.2–24.0)	<0.001
Anterior spacing								
*No (ref)*								
Yes	−1.7 (−5.4–2.1)	0.383	4.6 (0.5–8.6)	0.027	3.6 (−5.4–12.6)	0.433	17.6 (9.0–26.1)	<0.001
Impacted teeth								
*No (ref)*								
Yes	−1.6 (−5.5–2.3)	0.422	−3.2 (−8.1–1.7)	0.203	−3.7 (−13.2–5.8)	0.444	−10.4 (−20.7 to −0.6)	0.049

Multivariable linear regression, adjusted for other malocclusion traits, age group, country of origin, municipality type, parental income, parental education, and caries. Analyses restricted to individuals with malocclusion and complete data (*n* = 520). Intercepts omitted from the table for clarity. CPQ, Child Perceptions Questionnaire (CPQ_11–14_–ISF:16); PIDAQ, Psychosocial Impact of Dental Aesthetics Questionnaire.

## Discussion

This study examined the associations between separate malocclusion traits and OHRQoL among adolescents with malocclusion. Further, potential sex differences in OHRQoL and effect modification by sex was investigated. Anterior crowding showed the most consistent and robust association with poorer OHRQoL in both instruments. Although females reported a higher overall burden of OHRQoL impacts, there was limited evidence of effect modification by sex in the associations between separate malocclusion traits and OHRQoL.

In the trait-specific analyses, anterior crowding showed the most consistent association with poorer OHRQoL in both instruments, whereas spacing was associated with poorer OHRQoL only when assessed using the orthodontic-specific PIDAQ. Both anterior crowding and spacing are visible malocclusions located in the esthetic zone, which may explain their stronger impact on perceived OHRQoL.

In the unadjusted analyses, patients with impacted teeth reported better OHRQoL than those with other malocclusion traits; however, this finding did not persist after adjustment. This may partly reflect age-related differences, as patients with impacted teeth are typically referred at a younger age, while specialist orthodontic treatment for other malocclusion traits is often deferred until the permanent dentition is fully erupted. Taken together, these findings suggest that clinically severe malocclusions such as impacted teeth may not always be perceived as highly burdensome by adolescents, underscoring the importance of clinician–patient communication when discussing treatment need.

Adolescents with reverse overjet reported better OHRQoL than those with other malocclusion traits, particularly when assessed with PIDAQ. Although this association remained significant after adjustment, it should be interpreted with caution given the small subgroup size (*n* = 14).

Malocclusion traits frequently co-occur, and adolescents often present with more than one deviation simultaneously. In unadjusted analyses, observed associations between separate malocclusion traits and OHRQoL may therefore partly reflect the influence of co-existing malocclusions. To address this, the adjusted models included all malocclusion traits simultaneously, allowing the associations to be interpreted as independent of other concurrent deviations. This approach strengthens the trait-specific interpretation of the findings, particularly for anterior crowding, which remained consistently associated with poorer OHRQoL after mutual adjustment.

In the malocclusion group, females consistently reported poorer OHRQoL compared with males, as reflected by the higher CPQ and PIDAQ scores. These results are consistent with earlier literature highlighting the same relationship [6, 27, 37, 38]. Previous research has also shown that females have a greater self-perceived orthodontic treatment need and are more dissatisfied with their teeth than males [16, 17]. These sex differences may explain why, despite malocclusion occurring with similar frequency in both sexes, females are consistently overrepresented in orthodontic populations [1, 2, 13–15]. However, in the stratified analyses, the associations between separate malocclusion traits and OHRQoL were largely similar in males and females, indicating no clear effect modification by sex. This suggests that although females generally report a higher overall burden of OHRQoL impacts, the relative influence of separate malocclusion traits on OHRQoL does not appear to differ substantially between sexes.

With few exceptions, CPQ and PIDAQ showed largely consistent patterns across malocclusion traits. One exception was observed in the adjusted analyses: reverse overjet was associated with better OHRQoL and spacing with poorer OHRQoL, but only when assessed with the orthodontic-specific PIDAQ. Corresponding CPQ estimates pointed in the same directions but were not statistically significant. Overall, however, the similarity in findings suggests that CPQ is sufficiently sensitive to capture OHRQoL impacts in adolescents with malocclusion, despite being a generic instrument. Future studies directly comparing the generic CPQ and orthodontic-specific PIDAQ, including assessments of correlation and responsiveness to change over time, are warranted to further inform instrument choice in orthodontic research.

To ensure methodological consistency across study sites, malocclusion assessments were performed on a standardized intraoral photograph series (five views) and dental records rather than direct clinical examinations. This approach enabled a single examiner, calibrated prior to study initiation, to conduct all malocclusion assessments, despite patient examinations being carried out by several investigators. This methodology is supported by evidence showing that assessments based on standardized orthodontic intraoral photograph series have comparable validity to assessments based on photographs in combination with study models [39].

### Strengths and limitations

The multicenter design represents strength of this study, enhancing generalizability. Another important methodological strength is the trait-specific analytical approach, allowing the assessment of separate malocclusion traits rather than relying on a broad malocclusion versus no malocclusion comparison. Further strengths included that potential confounding was addressed through multivariable adjustment, including simultaneous adjustment for co-existing malocclusion traits as well as relevant demographic, socioeconomic and clinical confounders. Moreover, the use of both a generic and an orthodontic-specific OHRQoL instrument provided complementary perspectives on OHRQoL impacts. In addition, effect modification by sex was formally examined using interaction terms and sex-stratified analyses. A further advantage is the use of Swedish national registers with extensive coverage and high data quality [40–42].

A key limitation of the present study is the potential for selection bias arising from the use of different recruitment settings. Adolescents with malocclusion were recruited from orthodontic clinics and were actively seeking orthodontic treatment. Consequently, they may not be fully representative of the whole population of individuals with malocclusion, as treatment-seeking adolescents are likely to report poorer OHRQoL than those with malocclusion who do not seek orthodontic care. In contrast, the no malocclusion group was recruited from general dentistry clinics. Importantly, direct comparisons between the two groups were not an aim of this study. The no malocclusion group was included to provide a descriptive reference rather than a comparison group. The inclusion of a no malocclusion group was further motivated by the potential for future longitudinal follow-up, which would enable evaluation of changes in OHRQoL over time and assessment of the responsiveness of OHRQoL instruments in individuals with and without malocclusion.

While the clinic-based design represents a limitation, conducting truly population-based orthodontic studies is challenging. This is partly due to difficulties in identifying an optimal age for assessment. Older adolescents may already have initiated orthodontic treatment while younger adolescents may not yet experience a measurable impact on OHRQoL. These structural constraints limit the feasibility of representative sampling and motivated the use of a clinic-based study design.

A further limitation is that some malocclusion traits were relatively infrequent, resulting in limited precision for these estimates.

### Clinical implications

The findings support a trait-specific assessment of malocclusion in OHRQoL studies, as different malocclusion traits appear to be associated with OHRQoL to varying degrees. Although females with malocclusion reported poorer OHRQoL than males with malocclusion, there was no evidence of sex modifying the associations between separate malocclusion traits and OHRQoL, and the findings do not support sex-specific weighting of malocclusion traits in clinical assessment. From a measurement perspective, the largely consistent patterns observed in both the generic and orthodontic-specific instruments suggest that generic instruments may be adequate for capturing OHRQoL impacts in orthodontic populations.

## Conclusions

Among adolescents with malocclusion, females report poorer OHRQoL than males. Anterior crowding shows the strongest association with poorer OHRQoL. Sex shows limited effect modification in the associations between separate malocclusion traits and OHRQoL.

## Supplementary Material

cjag032_Supplementary_Data

## Data Availability

Raw data and programming code are available from the corresponding author upon reasonable request and with the permission of SKaPa, Statistics Sweden, and the Swedish Ethical Review Authority. Restrictions apply.
