# Supplementary material for: Malocclusion traits and oral health-related quality of life in adolescents: a multicenter cross-sectional study
Source: Eur J Orthod. 2026 May 19;48(3):cjag032. doi: 10.1093/ejo/cjag032 (PMC13186198; doi:10.1093/ejo/cjag032)
Supplement: cjag032_Supplementary_Data [file cjag032_supplementary_data.zip › Supplementary Table S1 (1).docx]

**Supplementary Table S1.** Variables, data sources, and procedure for data cleaning.

| **Variable** | **Data source** | **Variable name  in registry** | **Data cleaning** |
| --- | --- | --- | --- |
| **Demographic variables** | | | |
| Sex | SCB/TPR | Kon | - |
| Year of birth | SCB/TPR | FodelseAr | - |
| Country of origin | SCB/TPR | UtlSvBakg | Categorized according to SCB definitions as having either a “foreign background” (individuals born abroad or born in Sweden to two foreign-born parents) or a “Swedish background” (individuals born in Sweden with at least one Swedish-born parent). |
| Municipality type | SCB/LISA | Kommun | The variable ‘Kommun’ (municipality) was measured at the year of study enrolment and classified according to DEGURBA (degree of urbanization) based on the SCB ‘kn_kopplingar’ dataset (2023) into “urban” (cities) and “non-urban” (towns, suburbs, and rural areas). |
| **Socioeconomic variables** | | | |
| Linkage to (adoptive) parents | SCB/MGR | - | Parental data were obtained from the biological mother and father or, when applicable, from adoptive parents or legal custodians. No imputation was performed. |
| Parental income | SCB/LISA | DispInkKe04 | Net yearly income (equivalized for household size) for the study enrolment year was calculated as the mean of both parents’ disposable incomes. If data were available for only one parent, the reported mean was based on that parent alone. If enrolment-year data were unavailable, values were substituted with the preceding year; if both years were unavailable the value was set to missing. No imputation was performed. Income levels were compared with the national median for the corresponding year: 2021: 278255 SEK, 2022: 292007 SEK and 2023: 312464 SEK (source SCB). For analysis, parental income was dichotomized: low economic standard was defined as disposable income < 60% of the national median (SCB definition), and middle/high as ≥ 60%. |
| Parental education | SCB/LISA | Sun2000Niva_Old | Parental highest grade completed for the year of study enrolment. The highest level attained by either parent was used as a proxy for household educational level. If both parents lacked data on education, data was categorized as missing.  Educational level (Sun2000Niva_Old; 7 levels) was condensed into three categories: primary/lower secondary (levels 1–2), upper secondary (levels 3–4), and university/college education (levels 5–7). |
| **Caries** | | | |
| DFT | SKaPa | DFT | The variable DFT (permanent teeth) was extracted for the year of study enrolment. Baseline was defined as the value from the questionnaire year; if missing, values from y−1, y+1, y−2, y+2, y−3, y+3, and y−4 were used in that order. If missing for all those years, data was considered missing. |
| **Occlusal status** | | | |
| Malocclusion assessment IOTN-DHC | Intraoral photographs and dental records | - | Malocclusion assessment was based on a standardized series of intraoral photographs (five views: frontal, right and left buccal, maxillary occlusal, and mandibular occlusal), supplemented with information extracted from patient dental records. Extracted data included overjet measurements, lip competence (complete vs. incomplete closure), the presence of anterior or lateral functional mandibular shifts, and dental radiographs.  The dental health component of the Index of Orthodontic Treatment Need (IOTN-DHC) was used to classify overall orthodontic treatment need and to categorize participants as having malocclusion (IOTN-DHC grades 3–5) or no malocclusion (IOTN-DHC grades 1–2). |
| **Oral Health Related Quality of Life** | | | |
| PIDAQ & CPQ | - | - | Item-level missing data were handled using within-person median imputation. Specifically, missing responses within each questionnaire were replaced with the median of the available items for the same individual, conditional on the proportion of missing data not exceeding 10 items in total across both instruments. For the PIDAQ, imputation was based on the individual’s median across all PIDAQ items, whereas for the CPQ it was based on the median of items 3–18. All 18 CPQ items (2 global and 16 domain-specific) were included in the imputation process. Participants with more than 10 missing responses across the combined set of PIDAQ and CPQ items were excluded from further analyses (per-protocol exclusion). In total, 40 participants required imputation, and 10 participants were excluded due to exceeding the missing data threshold. |

**Abbreviations**

DEGURBA Eurostat’s Degree of Urbanization
DFT Decayed, Filled Teeth (permanent)
IOTN-DHC Index of Orthodontic Treatment Need, Dental Health component
LISA Longitudinal Integrated Database for Labor Market and Health Insurance Studies
MGR Multi Generation Register
PIN Personal identification number
SCB Statistics Sweden
TPR Total Population Register
SKaPa Swedish Quality Registry for Caries and Periodontal Disease
