# Supplementary material for: Malocclusion traits and oral health-related quality of life in adolescents: a multicenter cross-sectional study
Source: Eur J Orthod. 2026 May 19;48(3):cjag032. doi: 10.1093/ejo/cjag032 (PMC13186198; doi:10.1093/ejo/cjag032)
Supplement: cjag032_Supplementary_Data [file cjag032_supplementary_data.zip › Supplementary Figure S1 (1).pdf]

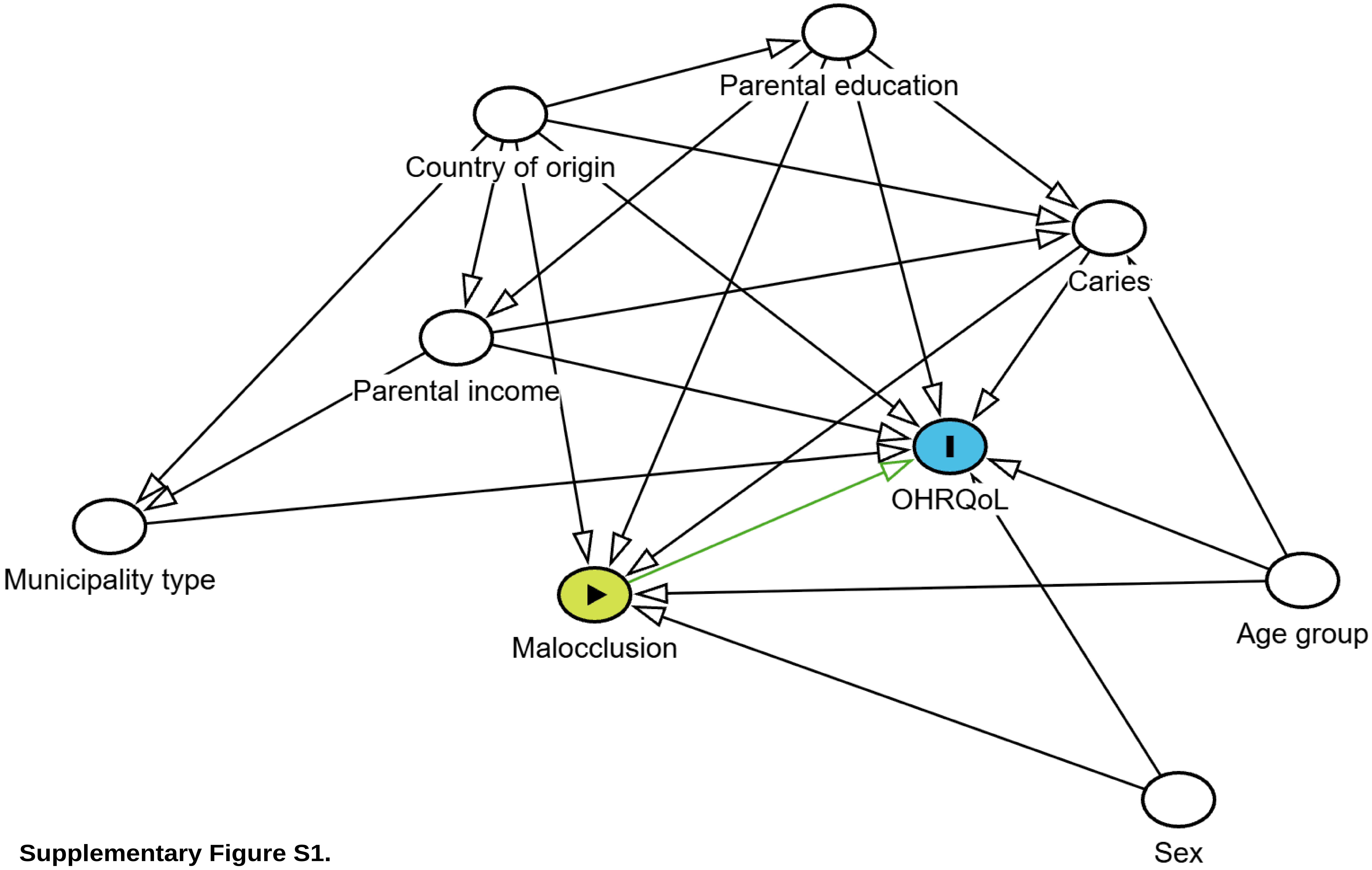

Supplementary Figure S1.
Directed acyclic graph (DAG) illustrating the assumed causal relationships between malocclusion and oral health-related quality of life (OHRQoL), including potential confounders.
